# Supplementary material for: Genomic diagnosis for children with intellectual disability and/or developmental delay
Source: Genome Med. 2017 May 30;9:43. doi: 10.1186/s13073-017-0433-1 (PMC5448144; doi:10.1186/s13073-017-0433-1)
Supplement: Supplementary file 1 — Supplemental Methods [43, 44]. (DOCX 124 kb) [file 13073_2017_433_MOESM1_ESM.docx]

**SUPPLEMENTAL METHODS**

**Study participant population**

There was no public recruitment for this study. Criteria for study inclusion was for the participants to have a clinical relationship with the pediatric neurologist or medical geneticist listed as co-investigators for the study. Individuals who presented with mild to severe ID were considered for study enrollment if their condition could not be accounted for by known causes (such as inborn errors of metabolism, lysosomal storage or mitochondrial disorders, Fragile X-associated mental retardation, Rett syndrome or other neurodegenerative conditions, Prader-Willi syndrome, or severe and documented birth asphyxia). Some of the participants exhibited autistic features and other behavioral abnormalities, but these characteristics had to be found in conjunction with other discrete DD/ID phenotypes prior to enrollment. The study physicians offered inclusion to individuals based on the following neuropsychometric criteria: severe ID - measurable IQ less than 40, usually non-ambulatory, nonverbal, the most severely impaired subjects; moderate ID - IQ range 40 - 60, typically have delays affecting other systems as well, broad range of delays/disabilities; mild ID - IQ range 60 - 70, trainable, may have delays affecting other systems. In the absence of neuropsychometric testing (i.e. IQ), decisions regarding study inclusion, and severity of ID, were deferred to the pediatric neurologist or geneticist.

**Filtering**

*De novo* variants were identified as heterozygous calls in the proband, and we required that the proband and their parents each had a read depth of 10X, at least 20% of proband reads contained the alternate allele, less than 5% of parental reads contained the alternate allele, and a minor allele frequency (MAF) ≤1% in 1000 Genomes [43], EVS [44] and ExAC [15]. Variants were also restricted to those where two or less alternate alleles were identified, the batch allele count was one, there were less than 40 counts in an internal allele frequency database, and the VQSR Filter class was not “Low Quality”. All candidate *de novo* variants that affected a protein, were within 10 nt of a splice site, or had a scaled CADD score ≥10 [21] were manually reviewed. Variants were similarly identified using X-linked, compound heterozygote, and recessive inheritance filters. No specific gene lists were used for identifying DD/ID variants in the proband.

In cases where one or both parents were unavailable, rare, potentially damaging variants were identified in the proband as heterozygous calls with a read depth of 10X, at least 20% of proband reads contained the alternate allele, and a minor allele frequency ≤0.1% in 1000 Genomes [43] and ExAC[15]. Variants were further restricted to those that affected a protein, were within 10 nt of a splice site, or had a scaled CADD score ≥20[21]. When available, parent read depth was required to be 10 reads, with less than 5% of reads representing the alternate allele. Variants were similarly identified using X-linked, potential compound heterozygote and recessive inheritance filters in probands with one or no parents available.

Three gene lists were employed for secondary variant identification in parent participants. Variants meeting the above guidelines in genes included on the ACMG gene list[14] were reviewed. Variants with recessive inheritance in genes that are associated with disease in OMIM[13] were also reviewed. Lastly, carrier status was assessed for three genes: *CFTR*, *HBB*, and *HEXA*.

Secondary variants were identified in parents as non-reference calls in which there were two or less alternate alleles, a batch allele frequency of ≤10, <40 counts in an internal allele frequency database, and the VQSR Filter class was “PASS”. Variants were also restricted to those that either affected a protein, a splice site, or had a scaled CADD score ≥15[21]. Note that for the ACMG[14] and OMIM[13] gene lists, a minor allele frequency of ≤1% in 1000 Genomes [43] and ExAC [15] was used, while for the carrier gene list, a minor allele frequency ≤25% in 1000 Genomes [43] and ExAC[15] was used.

Rare variants (minor allele frequency of ≤1% in 1000 Genomes, EVS and ExAC [15, 43, 44]) that have been submitted to the ClinVar database[6] as pathogenic or likely pathogenic (or conflicting reports of pathogenicity, but at least one report as pathogenic or likely pathogenic) were also manually reviewed in each family.

**Variant classification**

The criteria used to evaluate the clinical relevance of sequence variants were similar to those stated by Richards and colleagues in the recently published ACMG recommendations [20]. Variants are classified into one of five categories: pathogenic, likely pathogenic, uncertain significance, likely benign or benign.

Below is a brief summary of variant properties used to support any given designation. Consistent with ACMG rules, multiple lines of evidence are required to support assignments of pathogenic or likely pathogenic, while classifications of benign or likely benign require allele frequency estimates too high to be plausibly connected to disease and/or computational predictions that a variant is benign. Conflicting or ambiguous lines of evidence generally result in a designation of VUS.

Pathogenic: 1) Previous reports that a variant is pathogenic, where primary data is available for analysis, and the interpretation of pathogenicity is still supported (by allele frequencies, mechanism of action, other scientific literature, etc.). 2) Variant clearly results in loss-of-function in a gene where loss-of-function is a known mechanism of disease. 3) Variant is missense and computationally predicted to be damaging, in a gene where missense variation is a known mechanism of disease. 4) Variant is *de novo* and predicted to be damaging or loss-of-function in a well-established dominant disease gene. 5) Variant(s) are recessive or compound heterozygous (established via parental assessment), at frequencies low enough to be plausible for disease, predicted to be damaging, and present in a gene known to be recessively associated with disease. 6) Patient’s observed phenotype(s) is specifically consistent with those reported for the associated disease.

Likely pathogenic: Variants that meet the criteria for Pathogenic, but have one of the following inconsistencies or uncertainties – 1) Uncertainty regarding the molecular effect of the variant (e.g. a variant resulting in a premature stop near the end of a protein, affecting non-canonical splice sites, or affecting splicing in only a subset of transcript isoforms). 2) Patient’s observed phenotype(s) are uncertain, undefined, or somewhat inconsistent with those reported for the associated disease. 3) There is a discrepancy between the type of variation and known mechanism of disease. 4) Inheritance status is unknown.

VUS: 1) Variant is *de novo* and computationally predicted to be damaging. 2) Variant(s) are very rare, predicted to be damaging, and exist in compound heterozygous or recessive states. 3) Variant impacts a gene with a specific, plausible biological connection to disease. 4) Variant impacts a gene predicted to be generally intolerant to variation. 5) Any variant for which there are lines of evidence supporting both pathogenic and benign interpretations.

Likely Benign: 1) Variant impacts a gene predicted to be tolerant of variation. 2) Variant has an allele frequency (in external or internal databases) near the observed frequency of disease. 3) Variant is predicted computationally to be benign (e.g., synonymous replacements, lack of conservation, etc.).

Benign: Variant has an allele frequency (in external or internal databases) that is higher than the observed frequency of disease.

**Return of results**

After the results were analyzed, participants were scheduled for a return of results conversation with a medical geneticist and certified genetic counselor. Variants of interest were discussed in a private setting and clinical significance of findings were addressed along with any questions posed by study participants. Genetic counselors provided each family with a document detailing information about variants identified in their sample(s) and information about support groups, specialty clinics, or other resources relevant to their specific finding(s).

**Reanalysis of exomes and genomes**

In December 2015, variants from all analyzed families were reannotated and refiltered. Updated annotations included: ClinVar [6] (accessed 12/3/2015, clinvar_20151201.vcf), ExAC release r0.2 [15], data from DDG2P [16] (<https://decipher.sanger.ac.uk/ddd#ddgenes>, accessed November 18, 2014 and December 3, 2015; ddg2p_20141118 and ddg2p_20150701), and data from several publications[17-19].

Filtering for primary variants was performed as described above. One additional, less-stringent *de novo* filter was also employed, which removed the depth requirement for all members of the trio. Filters were also utilized to identify rare variants submitted to ClinVar [6] as pathogenic or likely pathogenic.

**Analysis of trios as singletons**

For all families that underwent WGS, we used family-specific VCFs that were pre-filtered for rare variants (≤ 2 alternate alleles, ≤ 1% MAF in EVS [44], ExAC[15] and 1000G [43], batch allele count ≤10, ≤40 counts in an internal allele frequency database, CADD ≥10 or protein-altering, or splice region, and exclude VQSR Low Quality) and then systematically decremented the batch allele count (AC) to reduce the allele count based on alleles contributed by parents. Using these AC-decremented files, we then filtered each proband (individually) for rare variants or compound heterozygous candidate variants.

Rare variants were defined as those where the proband was not homozygous reference, restricting to those with two or less alternate alleles, batch allele counts of one, ≤ four counts in an internal allele frequency database, EVS [44], ExAC Global allele frequencies [15] of ≤ 0.01% (≤0.2% each subpopulations), 1000G Global allele frequency [43] of ≤0.2% (≤0.5% in AA and EUR subpopulations), and LowQuality VQSR Filtered variants were excluded. Additional variations included restrictions based on CADD scaled scores of ≥10 or 15 (unless protein-altering, or within 10 nt of a canonical splice site).

Rare potential compound heterozygous variants were defined as those where the proband had two heterozygous calls in the same gene, restricting to those with two or less alternate alleles, batch allele counts of ≤ 5, ≤ 40 counts in an internal allele frequency database, EVS [44], ExAC Global allele frequencies [15]of ≤ 0.01% (≤0.2% each subpopulations), 1000G Global allele frequency [43] of ≤0.2% (≤0.5% in AA and EUR subpopulations), and LowQuality VQSR Filtered variants were excluded.

Additional variations included restrictions to genes associated with disease in OMIM [13] or DDG2P[16], or restrictions based on RVIS score [28] (top 10% intolerant genes, top 20%, etc.).

Variants from these filter results were then ranked by CADD score[21]. In cases where we identified a returned variant (VUS, likely pathogenic or pathogenic), we calculated the CADD-based rank of that variant[21].

**RNA isolation**

2.5 mL of blood was collected in PAXgene RNA tubes (PreAnalytiX #762165) according to the manufacturer’s instructions and stored short-term at -20°C. RNA was isolated using a the PAX gene Blood RNA Kit (Qiagen #762164) according to the manufacturer’s instructions. Isolated RNA was quantified by Qubit^®^ (Thermo Fisher #Q32855).

**cDNA synthesis**

First strand synthesis of cDNA was performed from 250-500 ng of RNA using either Superscript™ III (Thermo Fisher #18080044) or Superscript™ IV VILO™ (Thermo Fisher #11766050) according to manufacturer’s instructions using either random hexamers or a mix of random hexamers and oligodT, with the exception that reverse transcription was carried out at 55°C for 20 minutes for Superscript™ IV VILO™.

***MTOR* PCR**

Amplicons were obtained by amplifying 3 ng of template cDNA using Phusion polymerase in HF buffer (NEB #M0531L) with 500 nM forward and reverse primers (IDT, Additional file 3: Table S7). Cycling conditions were as follows: (98°C, 30s), (98°C, 10s; 61°C, 30s; 72°C, 90s)x35, (72°C, 10m), (4°C, ∞).

***ALG1* PCR**

Amplicons were obtained by amplifying 30 ng of template cDNA using Phusion polymerase (NEB #M0531L) with 1 μM forward and reverse primers (IDT, Additional file 3: Table S7) and 3% DMSO. Cycling conditions were as follows: (98°C, 30s), (98°C, 10s; 58°C, 30s; 72°C, 90s)x7, (98°C, 10s; 60°C, 30s; 72°C, 80s)x7, (98°C, 10s; 62°C, 30s; 72°C, 70s)x7, (98°C, 10s; 64°C, 30s; 72°C, 60s)x7, (98°C, 10s; 66°C, 30s; 72°C, 50s)x7, (72°C, 10m), (4°C, ∞).

**ROCK2 western blot**

Live cells were collected using cell processing tubes (BD #362760), isolated according to the manufacturer’s instructions, and stored in liquid nitrogen in CTS™ Synth-a-Freeze^®^ Medium (Thermo Fisher # A13713-01) until use. Cell pellets were homogenized in RIPA buffer (1x PBS, 0.5% Sodium Deoxycholate, 0.1% SDS, 1% NP-40) supplemented with 1x cOmplete™ protease inhibitor cocktail (Roche #11697498001). Lysates were cleared at 500 x g for 5 minutes, then protein concentration was measured by Qubit^®^ (Thermo Fisher # Q33212) and lysates were equalized in concentration by dilution with lysis buffer. 100 µg of protein was loaded per lane, blots were blocked for 1 hour at room temperature in 5% milk in 0.05% PBS-T, then blots were probed with 1:250 rabbit anti-ROCK2 (N-terminal, Sigma-Aldrich HPA007459) or 2 μg/mL mouse anti-ROCK2 (C-terminal, Abcam ab56661) overnight at 4°C in 0.05% PBS-T. Blots were probed with β-actin as a loading control at 1:10,000 (Cell Signaling #8H10D10) for 1 hour at room temperature. Secondary antibodies were HRP-conjugated goat anti-rabbit IgG (Thermo Fisher #31460) and HRP-conjugated goat anti-mouse IgG (Thermo Fisher #31430). Signal was detected using an enhanced chemiluminescent substrate (Thermo Fisher #34095).

**qPCR**

Quantitative PCR was performed on cDNA synthesized as described above after being diluted 5 fold. 10 μl reactions were composed of 2 μl diluted cDNA, 5 μl Power SYBR Green 2X master mix (Applied Biosystems ref#4367659), 1.25 μl forward oligo (10 μM), 1.25 μl reverse oligo (10 μM), and 0.5 μl H_2_O. The oligos were designed to amplify a 50-70 bp fragment and the melting temperature of the product was used to verify the correct specificity of the oligos during each qPCR. At least two independent cDNA synthesis reactions were performed for each biological sample and the reactions were performed in quadruplicate technical replicates for each cDNA synthesis. The reactions were performed using Applied Biosystems QuantStudio 6 Flex. C_T_ values were obtained and used to calculate the ΔΔC_T_ values as a percentage of the affected individuals.
