# Supplementary material for: Genomic diagnosis for children with intellectual disability and/or developmental delay
Source: Genome Med. 2017 May 30;9:43. doi: 10.1186/s13073-017-0433-1 (PMC5448144; doi:10.1186/s13073-017-0433-1)
Supplement: Supplementary file 3 — Supplementary Figures S1–S5 and Tables S6 and S7. [45] (DOCX 2319 kb) [file 13073_2017_433_MOESM3_ESM.docx]

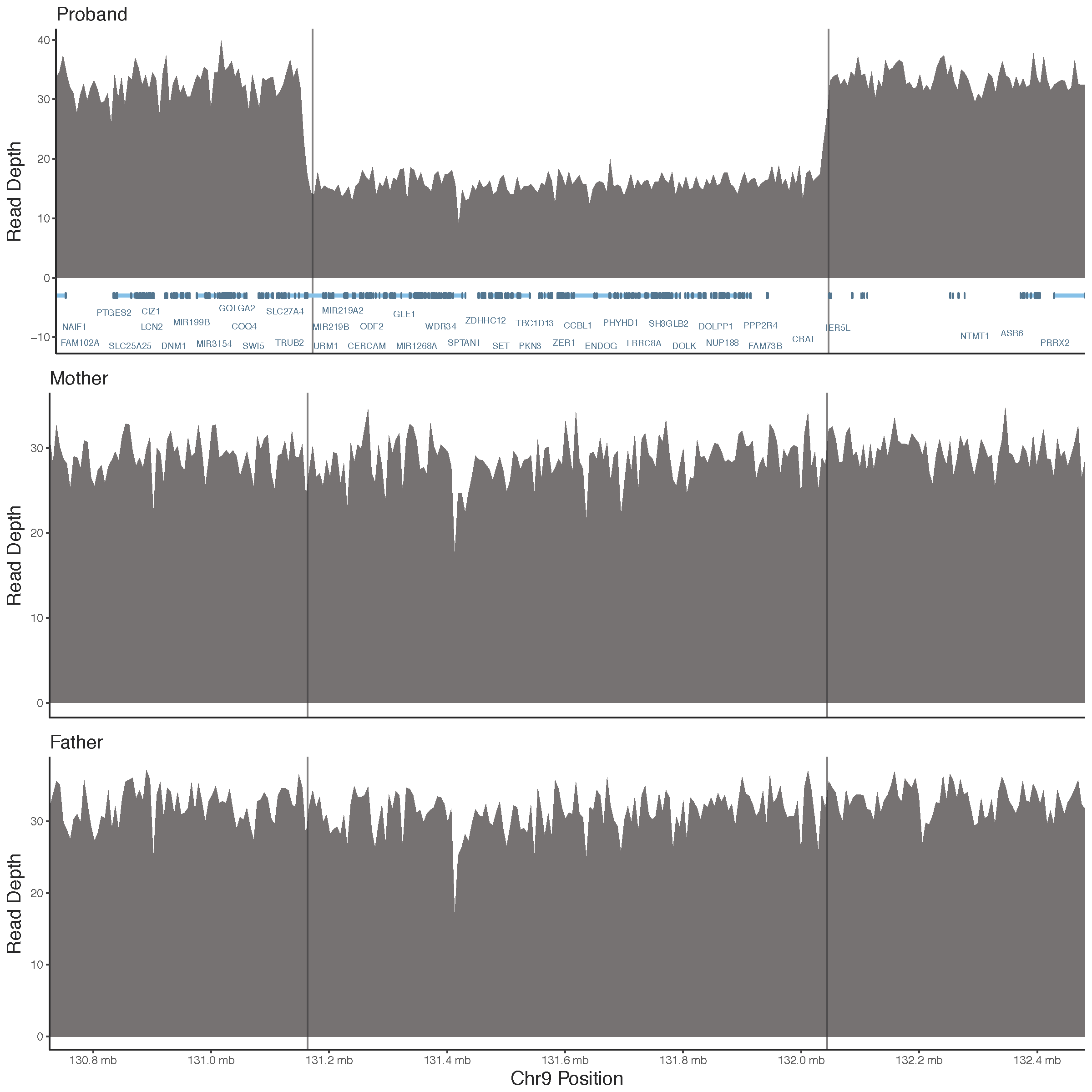


**Figure S1: Pathogenic *de novo* 9q34.11 deletion event identified by WGS.** Example of a pathogenic *de novo* heterozygous deletion called from WGS data. Read depths for the proband (00133-C), mother and father are shown. This 880-kilobase deletion event on chromosome 9 encompasses 24 annotated RefSeq genes [45] including *SPTAN1*, a known neurodevelopmental gene (MIM:613477).


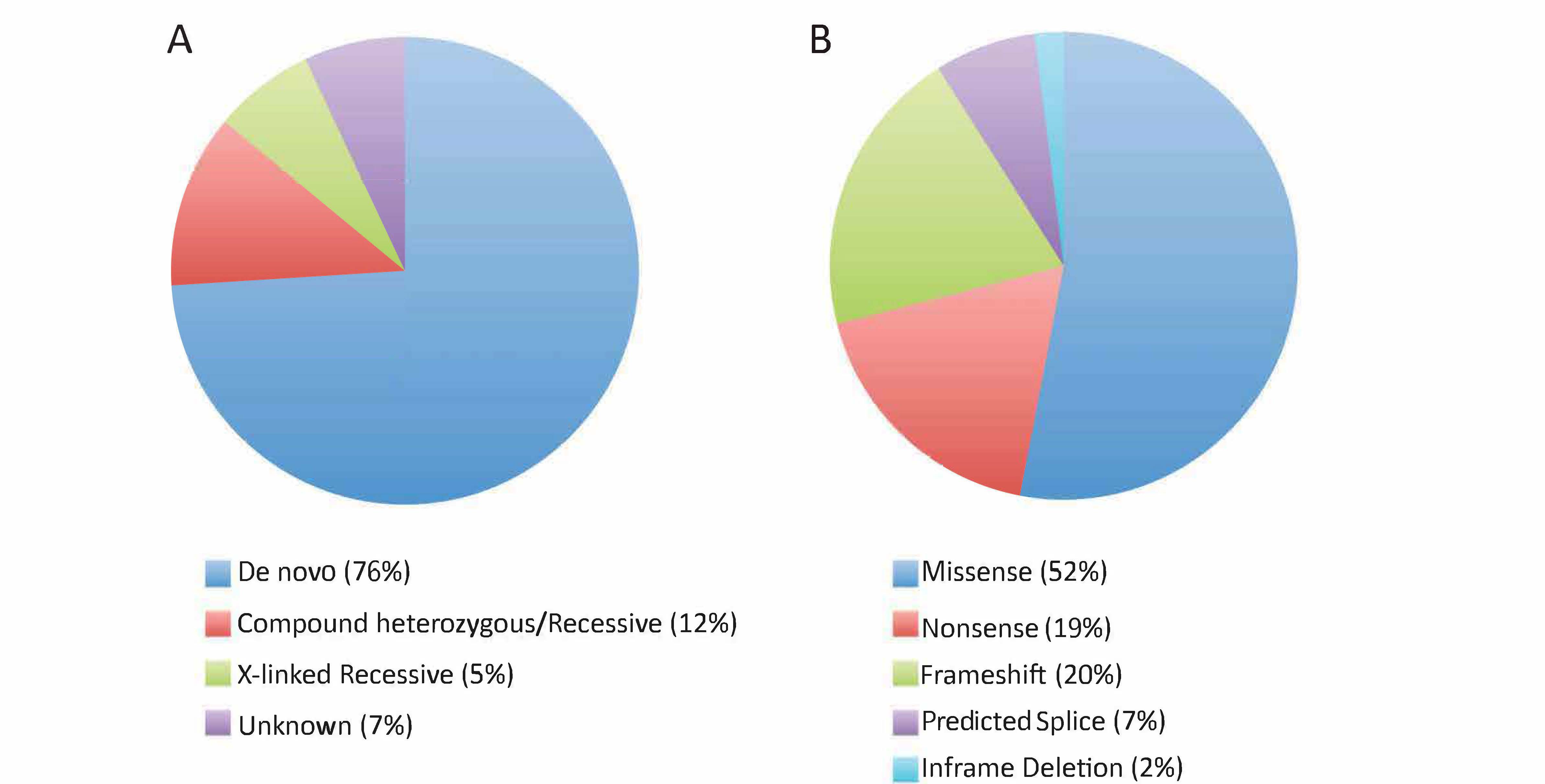


**Figure S2: Inheritance mechanism and molecular consequence of pathogenic/likely pathogenic SNVs returned to DD/ID-affected individuals.** (A) Inheritance pattern (expressed as a percentage) of variants returned to DD/ID-affected individuals who received a likely pathogenic or pathogenic finding. In the event that both parents were not available, inheritance could not be determined and this is represented by the term "unknown". (B) Mutation type (expressed as a percentage) of identified pathogenic/likely pathogenic variants. n=92 affected individuals.


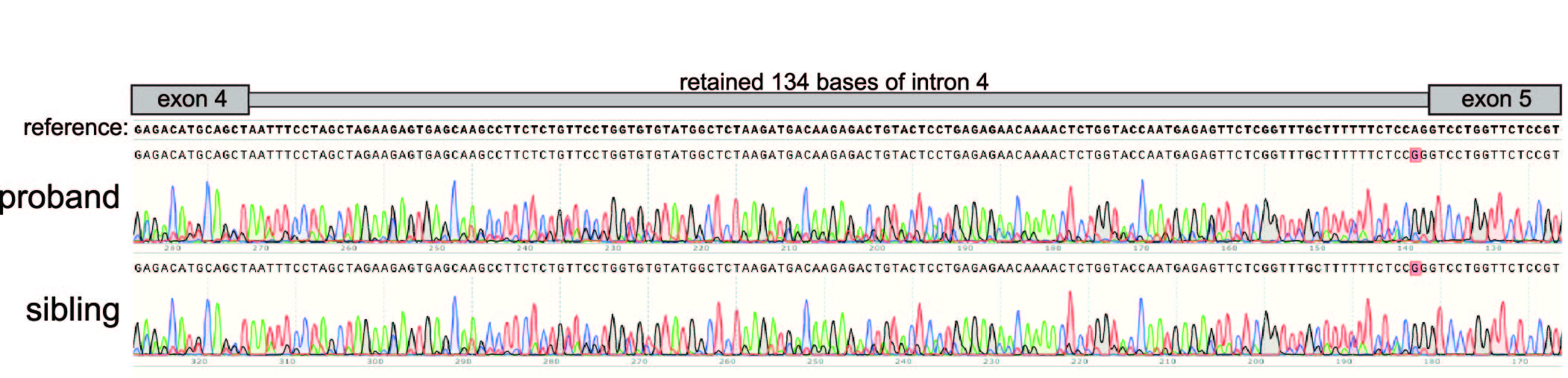


**Figure S3: The splice variant identified in *MTOR* leads to retention of 134 nucleotides of the 3’ end of intron 4 in the mRNA transcript.** Sanger sequencing confirmed the partial retention of intron 4 in cDNA synthesized from maternal half-siblings.


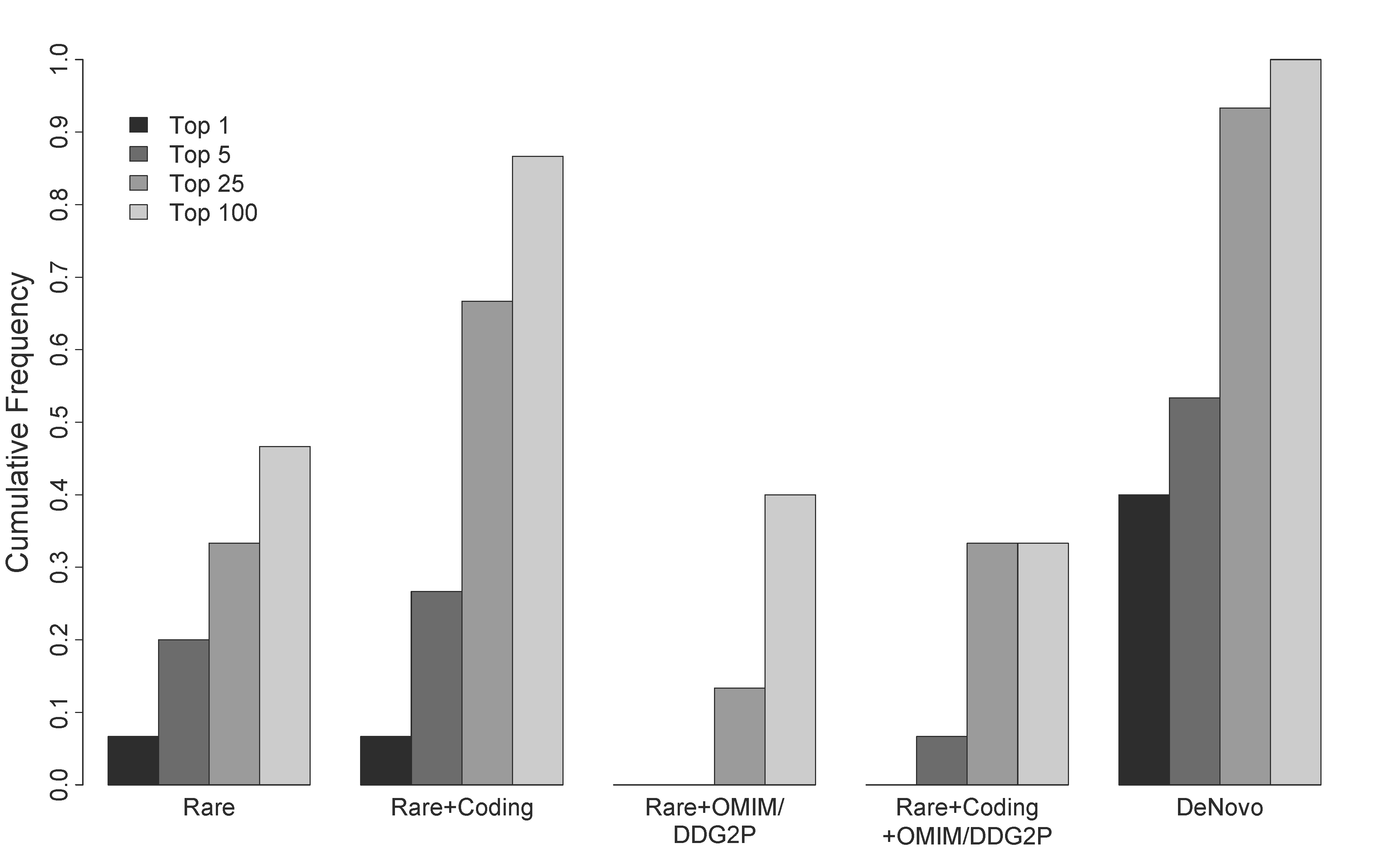


**Figure S4: Cumulative fractions (y-axis) of CADD-based ranks of variants of uncertain significance filtered without parental data relative to *de novo* events (“DeNovo”) defined with parental data.** VUSs are more difficult to identify without parental information, and restrictions to those variants affecting OMIM/DDG2P [13, 16] genes further limits the identification of these variants.


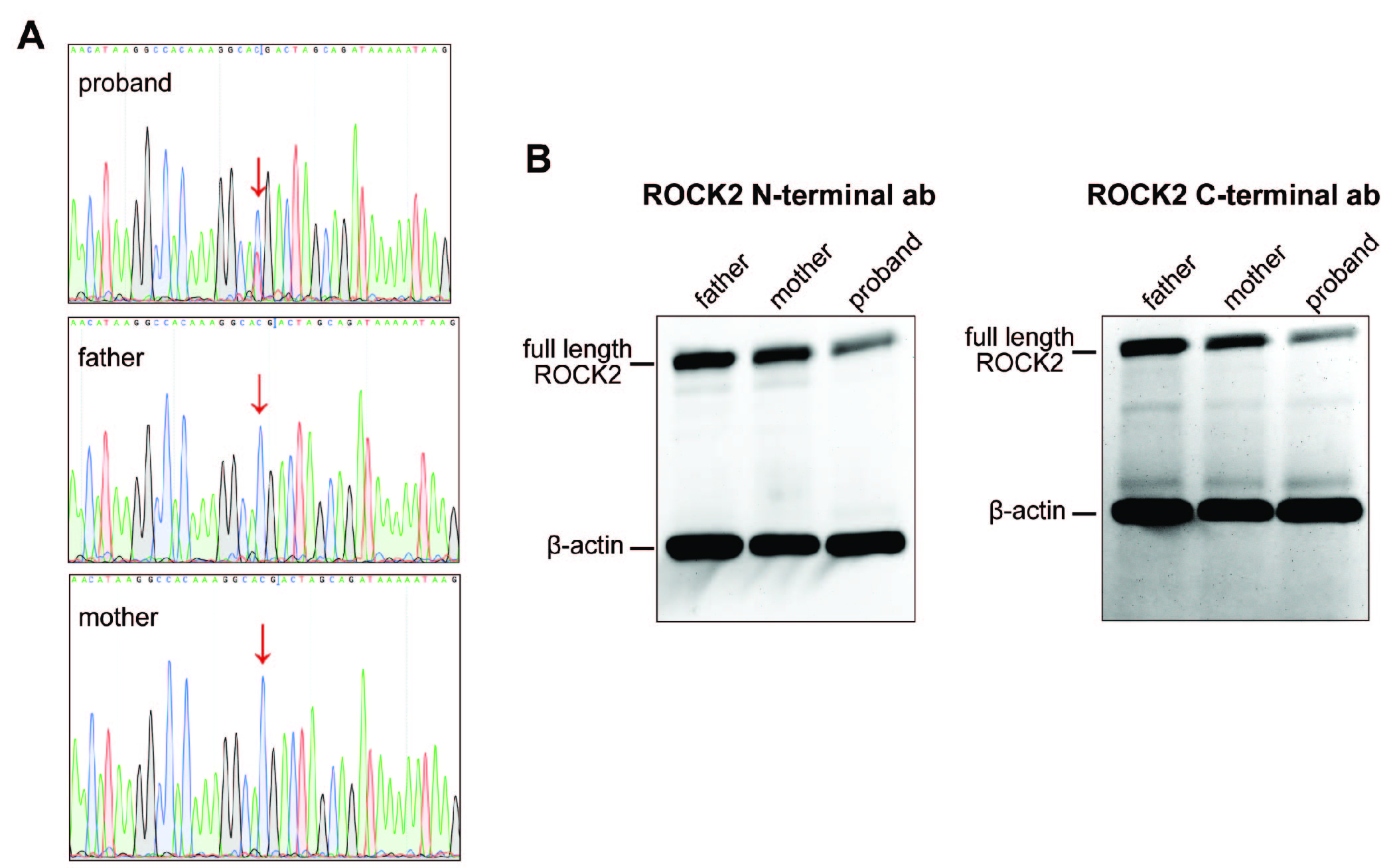


**Figure S5: mRNA is produced from the variant *ROCK2* allele, but truncated protein is not detected in the proband’s blood.** (A) Sanger sequencing from cDNA showed that the variant allele is transcribed and produces detectable mRNA. (B) Western blots were performed using antibodies directed against the N-terminus (Sigma-Aldrich HPA007459) and C-terminus (Abcam ab56661) from protein extracted from the proband, father, and mother. β-actin was used as a control (Cell Signaling #8H10D10).

|  |  | Mean | 1x cov | 4x cov | 8x cov | 20x cov | 30x cov |
| --- | --- | --- | --- | --- | --- | --- | --- |
| Exome | CCDS | 60.7X | 99.0% | 98.0% | 96.9% | 90.0% | 78.9% |
|  | Nimblegen v3 | 65.1X | 99.1% | 98.2% | 97.2% | 90.2% | 78.5% |
|  | ACMG genes | 57.5X | 99.4% | 98.9% | 98.1% | 91.5% | 79.0% |
|  | Gene Tests | 57.7X | 96.9% | 95.5% | 94.3% | 86.9% | 75.6% |
| Genome | CCDS | 37.1X | 99.6% | 99.6% | 99.5% | 96.5% | 78.4% |
|  | Nimblegen v3 | 37.4X | 99.6% | 99.5% | 99.4% | 96.1% | 78.0% |
|  | ACMG genes | 37.2X | 99.7% | 99.7% | 99.7% | 97.9% | 79.9% |
|  | Gene Tests | 35.9X | 99.4% | 99.4% | 99.3% | 93.4% | 73.9% |

**Table S6. Coverage metrics across 365 exomes and 612 genomes.**

Exome and genome coverage metrics across CCDS, Nimblegen exome v3 targets, 56 ACMG genes

and genes included as part of GeneTests (www.genetests.org; February 2015). For exomes,

n=365; for genomes, n=612.

Table S7. Oligos for quantitative PCR, PCR, and sequencing of ALG1, mTOR, and ROCK2 cDNA

| **gene** | **description** | **sequence (5' to 3')** |
| --- | --- | --- |
| GAPDH | Reverse | TTGATTTTGGAGGGATCTCG |
| GAPDH | Forward | ACGGGAAGCTTGTCATCAAT |
| ALG1 | Forward (E11:I11 junction) | GCCTGTGTGTGCTGTGAACT |
| ALG1 | Reverse (E11:I11 junction) | CCCAGAAGGATTTGGGTTCT |
| ALG1 | Forward (I11:E12 junction) | AGGCTCCCTTGGTTCTCTCT |
| ALG1 | Reverse (I11:E12 junction) | TCATGTTTCACCAGCTCATGT |
| MTOR | Forward (E4:I4 junction) | AATGAGGGCCGGAGACAT |
| MTOR | Reverse (E4:I4 junction) | GCTGTGCTCCTCCCTGTAGA |
| MTOR | Forward (I4:E5 junction) | TGAGAGTTCTCGGTTTGCTTT |
| MTOR | Reverse (I4:E5 junction) | GAAGAAGGTAGGGACGCTGA |

PCR oligos

| ALG1 | Forward (Exon 8:Exon 9 junction) | GCAGCTTTAGAAAAGTTTGAACAACTGA |
| --- | --- | --- |
| ALG1 | Reverse (Exon 13) | TTCCGGAACTGGTTTAGCTTGC |
| MTOR | Forward (Exon 2:Exon 3 junction) | TCCGAGAGATGAGTCAAGAGGAGT |
| MTOR | Reverse (Exon 6:Exon 7 junction) | CTTCTCTCAGACGCTCTCCCTC |

Sequencing oligos

| ROCK2 | Exon 17 | TCAGGAGAGATTTACTGATTTGGA |
| --- | --- | --- |
| MTOR | Reverse (Exon 5) | ACGCTGGGTTGTGAGAATCAGA |
| MTOR | Reverse (E5:E6 junction) | ATGTGTGCCTGTACCACTGAGG |
