# Supplementary material for: Genomic diagnosis for children with intellectual disability and/or developmental delay
Source: Genome Med. 2017 May 30;9:43. doi: 10.1186/s13073-017-0433-1 (PMC5448144; doi:10.1186/s13073-017-0433-1)
Supplement: Supplementary file 4 — Recurrent gene findings across affected 371 DD/ID-affected individuals. (DOCX 168 kb) [file 13073_2017_433_MOESM4_ESM.docx]

| Table S2. Recurrent gene findings across 371 DD/ID-affected individuals | | | | | |
| --- | --- | --- | --- | --- | --- |
| Gene | **Function** | **Associated clinical syndromes**  **(MIM number)** | **No. of unrelated individuals** | **Mutation type** | **Inheritance pattern** |
| MTOR | Protein kinase; mediates cell responses to stresses such as DNA damage and nutrient deprivation | Smith-Kingsmore syndrome (616638) | 4 | Missense (3); Splice | De novo (3); Unknown |
| SCN1A | Na channel; generation and propagation of action potential in neurons and muscle | Generalized epilepsy with febrile seizures (604403);  Epileptic encephalopathy, early infantile 6 (607208) | 3 | Frameshift; Nonsense; Intronic | De novo |
| FOXG1 | Transcriptional repressor; involved in brain development | Rett Syndrome, congenital variant (613454) | 3 | Missense (2); Frameshift | De novo |
| MECP2 | Transcriptional repressor; involved in embryonic development | Rett syndrome (312750) | 3 | Frameshift; Splice; Nonsense | De novo |
| SLC2A1 | Glucose transporter in mammalian blood-brain barrier | GLUT1 deficiency syndrome, infantile onset (606777); childhood onset (612126); Dystonia 9 (601042) | 3 | Splice; Missense; Frameshift | De novo |
| ANKRD11 | Inhibits ligand-dependent activation of transcription | KGB syndrome (148050) | 2 | Nonsense; Frameshift | De novo |
| ARID1B | Cell-cycle activation; component of SWI/SNF chromatin remodeling complex | Coffin-Siris syndrome 1 (135900) | 2 | Nonsense | De novo |
| ARX | Transcriptional repressor activity; involved in CNS development | Partington syndrome (309510); Proud syndrome (300004); Epileptic encephalopathy, early infantile 1 (308350); Hydranencephaly with abnormal genitalia (300215); Lissencephaly (300215); Mental retardation, X-linked 29 and others (300419) | 2 | Frameshift; Missense | De novo; Inherited |
| ASXL3 | Transcriptional regulator | Bainbridge-Ropers syndrome (615485) | 2 | Nonsense; Missense | Unknown |
| CHD2 | Chromatin remodeling | Epileptic encephalopathy, childhood-onset (615369) | 2 | Frameshift | De novo |
| CREBBP | Plays critical roles in embryonic development, growth control, homeostasis by coupling chromatin remodeling to TF recognition | Rubinstein-Taybi syndrome 1 (180849) | 2 | Missense; Splice | De novo |
| DDX3X | ATP-dependent RNA helicase activity | Mental retardation, X-linked 102 (300958) | 2 | Nonsense | De novo |
| EBF3 | Involved in B-cell differentiation, bone development, and neurogenesis | Hypotonia, ataxia, and delayed development syndrome (617330) | 2 | Splice; Missense | De novo |
| HCFC1 | Control of the cell cycle and transcriptional regulation during herpes simplex virus infection | Mental retardation 3,  X-linked (309541) | 2 | Missense | De novo; Inherited |
| KIF1A | Anterograde motor protein | Mental retardation, AD 9 (614255) | 2 | Missense | De novo |
| NBEA | Neuronal post-Golgi membrane traffic | Implicated in autism | 2 | Nonsense; Missense | De novo |
| MED13L | Transcriptional coactivator; involved in early development of heart and brain | Mental retardation and distinctive facial features with or without cardiac defects (616789) | 2 | Frameshift; Missense | De novo |
| SATB2 | Transcription regulation and chromatin remodeling | Glass syndrome (612313) | 2 | Frameshift; Nonsense | Unknown;  De novo |
| SCN2A | Na channel; generation and propagation of action potential in neurons and muscle | Epileptic encephalopathy, early infantile (613721);  Seizures, benign familial infantile, 3 (607745) | 2 | Missense | De novo |
| SCN8A | Na channel; Membrane depolarization during action potentials in most electrically excitable cells | Epileptic encephalopathy, early infantile, 13 (614558); Seizures, benign familial infantile 5 (617080) | 2 | Missense | De novo |
| SYNGAP1 | Ras GTPase activation protein; component of PSD associated with NMDA receptors at synapses | Mental retardation, AD 5 (612621) | 2 | Splice; Frameshift | De novo |
| TCF4 | Transcription factor; may play a role in nervous system development | Pitt-Hopkins syndrome (610954);  Corneal dystrophy, Fuchs endothelial, 3 (613267) | 2 | Missense; Frameshift | Unknown;  De novo |
| WDR45 | Cell cycle progression, signal transduction, apoptosis, gene regulation | Neurodegeneration with brain iron accumulation 5 (300894) | 2 | Missense; Splice | De novo |
