# Supplementary material for: Genomic diagnosis for children with intellectual disability and/or developmental delay
Source: Genome Med. 2017 May 30;9:43. doi: 10.1186/s13073-017-0433-1 (PMC5448144; doi:10.1186/s13073-017-0433-1)
Supplement: Supplementary file 5 — Pathogenic/likely pathogenic rates across families of varying structure and phenotypic complexity. (DOCX 48 kb) [file 13073_2017_433_MOESM5_ESM.docx]

| Family Structure | Trio  (n=251) | Duo  (n=37) | Singleton  (n=13) | Total*  (n=301) |
| --- | --- | --- | --- | --- |
| Simplex (n=93) | 39.0% (31/80) | 30.7% (4/13) | 0.00% (0/0) | 37.6% (35/93) |
| Multiplex (n=123) | 20.6% (20/97) | 13.3% (2/15) | 18.2% (2/11) | 19.5% (24/123) |
| 2^nd^ & 3^rd^ degree  (n=85) | 28.3% (21/74) | 11.1% (1/9) | 0.00% (0/2) | 25.9% (22/85) |

**Table S3. Pathogenic/likely pathogenic rates across families of varying structure and phenotypic complexity**

*38 families were excluded due to limited or no family history
